# Can we rely on the multiplex ligation-dependent probe amplification method (MLPA) for prenatal diagnosis?

**Published:** 2014-04

**Authors:** Mir Davood Omrani, Faezeh Azizi, Masoumeh Rajabibazl, Niloufar Safavi Naini, Sara Omrani, Arezo Mona Abbasi, Soraya Saleh Gargari

**Affiliations:** 1 *Department of Clinical Genetics, Faculty of Medicine, Shahid Beheshti University of Medical Sciences, Tehran, Iran.*; 2 *Department of Clinical Biochemistry, Faculty of Medicine, Shahid Beheshti University of Medical Sciences, Tehran, Iran.*; 3 *Faculty of Medicine, Shahid Beheshti University of Medical Sciences, Tehran, Iran.*; 4 *Edward Via College of Osteopathic Medicine, Virginia, USA.*; 5 *Feto-Maternal Unit, Mahdieh Hospital, Shahid Beheshti, University, Tehran, Iran.*

**Keywords:** *MLPA*, *Prenatal screening*, *Common aneuploidies*

## Abstract

**Background:** The major aneuploidies that are diagnosed prenatally involve the autosomal chromosomes 13, 18, and 21, as well as sex chromosomes, X and Y. Because multiplex ligation-dependent probe amplification (MLPA) is rapid and non-invasive, it has replaced traditional culture methods for the screening and diagnosis of common aneuploidies in some countries.

**Objective:** To evaluate the sensitivity and specificity of MLPA in a cross-sectional descriptive study for the detection of chromosomal aneuploidies in comparison to other methods.

**Materials and Methods: **Genomic DNA was extracted from the peripheral blood samples of 10 normal controls and the amniotic fluid of 55 patients. Aneuploidies screening of chromosomes 13, 18, 21, X and Y were carried out using specific MLPA probe mixes (P095-A2). For comparison purposes, samples were also tested by Quantitative Fluorescent-PCR (QF-PCR) and routine chromosomal culture method.

**Results: **Using this specific MLPA technique and data-analyzing software (Genemarker v1.85), one case was diagnosed with 45, X (e.g. Monosomy X or Turner’s Syndrome), and the remaining 54 cases revealed normal karyotypes. These results were concordant with routine chromosomal culture and QF-PCR findings.

**Conclusion:** The experiment demonstrates that MLPA can provide a rapid and accurate clinical method for prenatal identification of common chromosomal aneuploidies with 100% sensitivity and 100% specificity.

## Introduction

Chromosomal abnormalities are often the origin of miscarriage and birth defects. The most common are autosomal aneuploidy (75%), polyploidy (13%), sex chromosome abnormalities (8%) and structural imbalance (4%) (1, 2). Trisomy of chromosome 21, 13, or 18, in addition to sex chromosome aneuploidy, account for 60-80% of aberrant fetal karyotypes identified in cultured amniotic fluid cells (3). Prenatal diagnosis is commonly suggested to all pregnant women who have an increased risk of carrying a child with a chromosomal abnormality. In these cases, the diagnostic process requires amniocentesis, which is highly invasive and often risky (4). Although traditional karyotyping is a powerful method that reveals a range of numerical and structural chromosomal abnormalities with high precision (99.4-99.9%), it requires fetal cell cultures, making this method time-consuming, labor-intensive, and expensive (5-7). The detection efficiency and precision of karyotyping may also be considered a disadvantage since it detects chromosomal abnormalities that may only hold ambiguous or mild clinical relevance. The latter can result in patient anxiety and emotional dilemmas regarding the continuation of pregnancy, even though the results are unclear or the suggested phenotype may prove to be comparatively mild (8, 9).

In developed countries, new molecular methods have become accessible for rapid aneuploidy detection of the most common chromosome abnormalities (aneuploidies of chromosomes X, Y, 13, 18 and 21). For example, in a number of prenatal centers, Quantitative Fluorescent PCR (QF-PCR) analysis is already being suggested to women undergoing invasive testing. Other centers carry out multiplex ligation-dependent probe amplification (MLPA) for the rapid detection of aneuploidies of chromosomes X, Y, 13, 18 and 21 in amniotic fluid cells (10, 11). MLPA is a new PCR-based method that differentiates the copy numbers of specific sequences of DNA. This technique applies two-part probes of unique length that, when hybridized to adjacent target sequences on genomic DNA, can be joined together by the enzyme DNA ligase. This permits the amplification of all target sites, using a single primer pair that is complementary to the two free ends which are common to all probes. 

The products are run on a capillary electrophoresis system and detached by size, so that each peak is the amplification product of a specific probe. Using a series of normalization calculations, copy numbers can be specified for each target sequence and thus, for each chromosome. MLPA is a quick high output method shown to be powerful in pre-clinical settings. It permits for relative quantification of up to 50 different target sequences with just one reaction. MLPA eschews the detection of abnormalities with uncharted clinical relevance. It is less labor-intensive and cheaper compared to karyotyping and FISH (1, 5). This pilot study was designed and carried out in effort to convince the policy makers and stakeholders of the Iranian healthcare system of the value of this technique for screening and diagnosis purposes at hospitals and pre-natal centers throughout the country. 

## Materials and methods

In this cross-sectional study, participants were 55 pregnant females who had chosen to undergo amniocentesis, for either advanced maternal age or increased risk following pre-natal screening, as referred by Shahid Beheshti's Mahdieh Women Hospital from 2012-2013. 

The age range of this sample population was between 22-39 years old. For each case, 15-20 ml amniotic fluid samples (without blood contamination) were collected. Meanwhile, the peripheral blood samples were collected from 10 unrelated, healthy female for use as the control sample, and set up of the MLPA reaction. Informed consent was explained and acquired from each case. The study was approved by the ethics committee of Shahid Beheshti University of Medical Sciences. Genomic DNA was extracted from peripheral blood by use of the salting out purification method. Amniotic fluid cell’s DNA were collected (5 ml/sample) using the QIAamp DNA Mini kit (Qiagen; Hilden, Germany). To check the quality of the obtained DNA, both samples were run on a 0.5% agarose gel and optical density (OD) was measured at 260 nm and 280 nm using a spectrophotometer (Biophotometer plus; Eppendorf, Germany). Moreover, the concentration of each DNA sample was standardized to 100ng/μL. 

**Figure 1:**
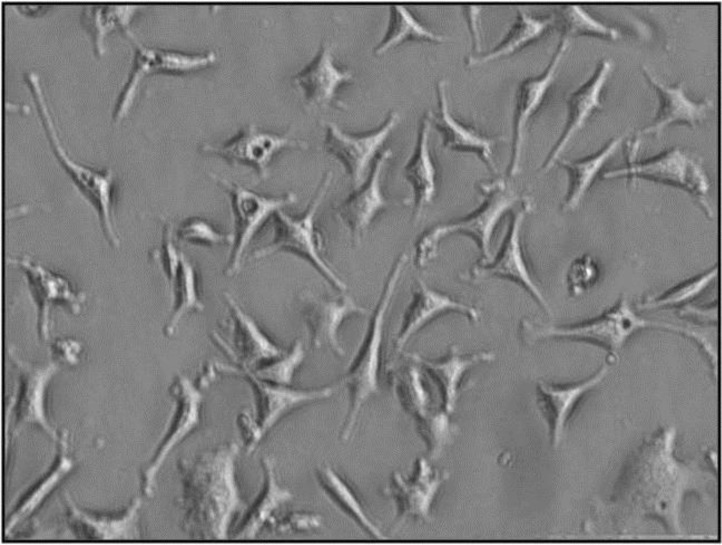
Image of amniotic fluid cells grown in Amniomax media after 15 days (Gibco, USA) ( Magnification 400X).To double check the results of MLPA obtained from the amniotic cells in the first day, they were grown and the experiment repeated with larger amount of cells and DNA

MLPA reagents (P095-A2 aneuploidy probe mixes) were purchased from MRC-Holland (Amsterdam, Kingdom of the Netherlands). The MLPA reaction was carried out using standard protocol. MLPA is not expected to detect low-grade chromosomal mosaicism. Peripheral blood cells or amniotic fluid cells (10-15 ml/sample) were cultured in accordance with the standard methods (Figure 1) (13). In addition, all samples were checked by QF-PCR method (11, 14-15). The results collected from MLPA, QF-PCR, and karyotyping were compared.

**Statistical analysis**

The data analyses of the MLPA tests were performed without the knowledge of karyotype results. The size and peak areas for each probe are quantified and processed by data-analysis software (Genemarker v1.85, Soft Genetics, LLC; State College, PA, USA) (12). Relative probe signals were assayed and compared with samples of normal male and female sex. In normal chromosome samples, the relative probe signal for all probes was expected as follows. A normal value is identified as a relative probe signal between 0.7 and 1.3. A relative probe value of <0.7 signifies a chromosome monosomy, while a relative probe value of >1.3 is indicative of 

chromosome trisomy.

## Results

**Figure 2:**
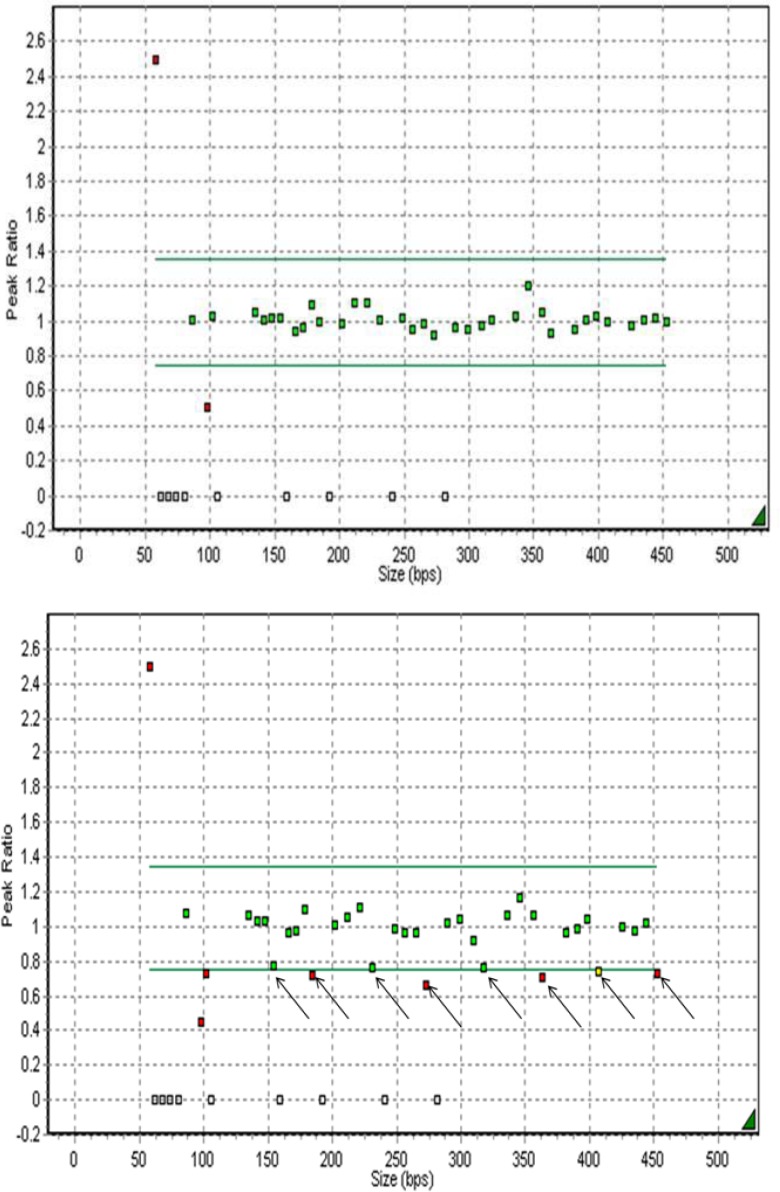
This is a part of the MLPA analysis report (Genemarker v1.85) of a normal disomic female and monosomic X female’s DNA, respectively. Normal relative probe signals are between the grey lines (0.7-1.3), and are depicted in green. Aberrant relative probe signals are depicted in red. The arrows indicate the eight chromosomes X signals, which all show a relative decrease in sample monosomic as compared to normal

In total, 55 amniotic fluid samples were tested with MLPA, QF-PCR, and karyotyping methods. In all samples, the results were in concordance with one another. In this study, one case with chromosome X monosomy [45, XO] was identified successfully by all methods. Figure 2 shows the abnormal copy numbers on chromosome X for one fetus sample. Diagnostic accuracy of MLPA was 1.0 (95% confidence interval (CI) 0.99-1.0) with a sensitivity of 100% (95% CI 0.96-1.0) and a specificity of 100% (95% CI 0.999-1.0).

## Discussion

The availability of quick, clear, and inexpensive genetic screening method for high-risk pregnancies is the reason for utilization of MLPA (16, 17). As of yet, two studies show that pregnant women prefer rapid aneuploidy recognition to karyotyping (1, 18). A Swedish survey showed that 70% of women preferred rapid testing to karyotyping. In the Netherlands, a nationwide prospective cohort study confirmed the accuracy of MLPA to detect aneuploidies of chromosomes 21, 13, 18, X and Y (5).

More studies by Gerdes *et al*, Van Opstal *et al* and Kooper *et al* added to the evidence for applicability of MLPA for detection of chromosomal aneuploidies in amniotic fluid (11, 14, 18, 19). At the level of public health, these investigations offer that rapid testing is the preferred strategy (5, 20-21). Compared to other methods accessible for rapid aneuploidy detection (RAD) [*i.e.* quantitative fluorescence polymerase chain reaction (QF-PCR) and fluorescence in situ hybridization (FISH)], MLPA has the advantage of detecting up to 50 loci in a single assay. Compared to FISH, MLPA is appropriate for high-output testing and is less expensive. Compared to QF-PCR, MLPA can easily be spread out to other genomic regions of known clinical relevance and can also be used as a highly effective method for the detection of sub-telomeric imbalances (22-27).

The MLPA technology entails ligation of probes corresponding to a chromosome-specific sequence that is unique within the genome. In contrast to polymorphic loci used for QF-PCR, these chromosome-specific sequences represent little or no variation, which avoids non-informativeness of the targeted sequences. Therefore, the MLPA method may be appropriate for combining speed and targeted testing of specific chromosomal inter- and/or intragenic regions. QF-PCR has the same inherent limitations as MLPA, in that it will not detect structural chromosome abnormalities; however, in contrast to QF-PCR, MLPA will not show 69, XXX triploidy (1, 28).

QF-PCR and MLPA are considered to be valid alternatives to karyotyping for specific referral reasons, albeit some clinically significant aberrations will remain unrecognized (1). Our study showed the successful use of MLPA for clinical molecular diagnosis with rapid and sensitive screening for chromosomal aneuploidies. Because processing and data analysis are completely automated, MLPA should be appropriate for large scale testing for chromosome aneuploidies in clinical diagnostic settings.

The purpose of previous studies was the substitution of rapid molecular techniques, such as MLPA, in place of traditional karyotype. In developing countries, prenatal diagnosis procedures were not well established and frequent studies needed to be carried out until stakeholders would accept it. In this study, we attempted to portray the positive results of these new techniques, in pursuit of applying them in educational hospitals.
